# Supplementary material for: Machine Learning-Assisted High-Content Imaging Analysis of 3D MCF7 Microtissues for Estrogenic Effect Prediction
Source: Res Sq. 2023 Oct 6:rs.3.rs-3343627. Preprint. [Version 1] doi: 10.21203/rs.3.rs-3343627/v1 (PMC10602099; doi:10.21203/rs.3.rs-3343627/v1)
Supplement: Supplement 1 [file NIHPPrs3343627v1-supplement-1.pdf]

Supplementary Files

This is a list of supplementary files associated with this preprint. Click to download.

- SupplementalData1.pdf
- SupplementalData2.pdf
- SupplementalData3.pdf
- SupplementalData4fullfeaturelist.xlsx
- SupplementalData5.pdf
- SupplementalData6DCCE2FeatureTable.csv
- SupplementalData7DCCPPTFeatureTable.csv
- SupplementalData8E2Top10Violinplot.pptx
- SupplementalData9PPTTop10Violinplot.pptx
- SupplementalData10E2PPTfeatureoverlap.csv
